# Insights from invasion ecology: Can consideration of eco-evolutionary experience promote benefits from root mutualisms in plant production?

**DOI:** 10.1093/aobpla/plz060

**Published:** 2019-09-23

**Authors:** Josep Ramoneda, Johannes Le Roux, Emmanuel Frossard, Cecilia Bester, Noel Oettlé, Beat Frey, Hannes Andres Gamper

**Affiliations:** 1 Group of Plant Nutrition, Department of Environmental Systems Science, ETH Zurich, Lindau, Switzerland; 2 Department of Biological Sciences, Macquarie University, Sydney, New South Wales, Australia; 3 South African Agricultural Research Council (ARC-Infruitec), Nieuwoudtville Northern Cape, Stellenbosch Central, Stellenbosch, South Africa; 4 Environmental Monitoring Group (EMG), Nieuwoudtville Northern Cape, South Africa; 5 Rhizosphere Processes Group, Swiss Federal Research Institute WSL, Birmensdorf, Switzerland; 6 Institute of Life Sciences, Scuola Superiore Sant’Anna, Pisa, Italy

**Keywords:** Adaptation, biological invasions, co-introduction, crop breeding, crop plant domestication, crop wild relative, ecological fitting, plant–microbe interactions, range expansion, root microbiomes

## Abstract

Mutualistic plant–microbial functioning relies on co-adapted symbiotic partners as well as conducive environmental conditions. Choosing particular plant genotypes for domestication and subsequent cultivar selection can narrow the gene pools of crop plants to a degree that they are no longer able to benefit from microbial mutualists. Elevated mineral nutrient levels in cultivated soils also reduce the dependence of crops on nutritional support by mutualists such as mycorrhizal fungi and rhizobia. Thus, current ways of crop production are predestined to compromise the propagation and function of microbial symbionts, limiting their long-term benefits for plant yield stability. The influence of mutualists on non-native plant establishment and spread, i.e. biological invasions, provides an unexplored analogue to contemporary crop production that accounts for mutualistic services from symbionts like rhizobia and mycorrhizae. The historical exposure of organisms to biotic interactions over evolutionary timescales, or so-called eco-evolutionary experience (EEE), has been used to explain the success of such invasions. In this paper, we stress that consideration of the EEE concept can shed light on how to overcome the loss of microbial mutualist functions following crop domestication and breeding. We propose specific experimental approaches to utilize the wild ancestors of crops to determine whether crop domestication compromised the benefits derived from root microbial symbioses or not. This can predict the potential for success of mutualistic symbiosis manipulation in modern crops and the maintenance of effective microbial mutualisms over the long term.

## Teaser

Plants establish beneficial symbiotic interactions with microbes that sustain plant nutrition and growth. These interactions are crucial for the stability of crop yields, particularly those of legumes. Likewise, in natural ecosystems symbioses facilitate the establishment and spread of invasive plant populations, to which microbes confer similar benefits as in crops. This manuscript builds on the parallelism between microbial symbioses in crops and invasive plants to develop a new framework. This framework can inform whether modern crops preserve the ability to obtain symbiotic benefits from microbes, and whether breeding programs could target symbioses for future plant production.

## Introduction

Rapid climate change and the need to utilize resources more efficiently call for crops that are able to cope with perturbation and stress while supporting stable yields. Root microbial mutualists such as arbuscular mycorrhizal fungi (AMF, phylum Glomeromycota; Tedersoo *et al*. 2019) and rhizobia (α- and β-Proteobacteria; Masson-Boivin and Sachs 2018) have the ability to benefit and increase crops’ access to additional nutrients and reduce abiotic and biotic stress. As a consequence, they improve the ability of crops to cope with increasingly unpredictable and changing abiotic and biotic conditions (Denison and Kiers 2011; Lau and Lennon 2012; Hurst 2017). For example, legumes have co-evolved with their microbial symbionts, particularly with rhizobia, which partly explains their ability to thrive under and adapt to both novel and rapidly changing environments (Porter *et al*. 2011; Sprent *et al*. 2017; Masson-Boivin and Sachs 2018). Likewise, some promiscuous legumes have taken advantage of interactions with unfamiliar rhizobia during range expansion to survive novel edaphic and climatic conditions (Simonsen *et al*. 2017). This is facilitated by the additional access to N, P, water and micronutrients afforded by rhizobia and AMF (Sprent *et al*. 2017). Microbial symbionts may also adapt to newly encountered abiotic conditions (e.g. soil pH; Dludlu *et al*. 2017) which, in turn, influence the performance and benefits received by host plants (Bennett and Klironomos 2019).

The symbiotic functioning of root-associated microbes can be drastically altered depending on host plant identity and local environmental conditions (Kiers *et al*. 2003; Martín-Robles *et al*. 2018; von Wettberg *et al*. 2018). This is important to bear in mind as crop domestication, breeding and cultivation are changing the genetic and ecological conditions under which plants are grown, which can compromise the benefits crops derive from microbial symbioses (Hetrick *et al*. 1992; Kiers *et al*. 2003; Sawers *et al*. 2018). For instance, cultivation under high mineral nutrient availability is expected to reduce allocation of photosynthetates towards symbiotic microbes (Kiers *et al*. 2003, 2007). The easy access to abundant soil resources for crops grown under high soil fertility can cause decreased dependency on mutualisms. These relaxed conditions may, in turn, lead to the accumulation of deleterious mutations in symbiosis-related genes in the plant genome. This can lead to compromised abilities of crops to recruit and reward microbial mutualists over the long run (Hetrick *et al*. 1992; Doebley *et al*. 2006; Kiers *et al*. 2007; Leff *et al*. 2017; von Wettberg *et al*. 2018). Additionally, selection for reduced investment into belowground as opposed to aboveground structures are known to decrease the benefits crop plants reap from mutualists such as mycorrhizae (Sawers *et al*. 2018).

The analogy between domesticated crops and invasive plants is remarkable. Both groups are the result of human-mediated translocation into novel environments. Invasive plants, like crops, often establish in open niche space with respect to competition and resource availability following physical disturbance (Gioria and Osborne 2014). At the same time, similar to domesticated crops, perturbation and stress due to changes in abiotic and biotic conditions pose challenges for the establishment and spread of non-native plants and impose strong selection pressures on them (Sakai *et al*. 2001). These selection pressures operate on narrow gene pools as many introduced species experience strong genetic bottlenecks (Barrett and Husband 1990; Hyten *et al*. 2006; Dlugosch and Parker 2008). It is also important to keep in mind that invasive plants experience stronger habitat filtering than crops, as the latter are supported by human intervention (i.e. weeding, fertilization, pest removal, active pollination and deliberate crossing and microbial inoculation). Lastly, like crops, invasive plants are often liberated from most macrofaunal and microbial associations from their native ranges. This is thought to play an important role in the success of many invasive plants, i.e. the lack of specialist enemies and antagonistic associations (Blossey and Nötzold 1995; Bossdorf *et al*. 2005). These parallels in the demographic and environmental conditions between plant invasions and agricultural settings, show that crop plant domestication, breeding and cultivation could learn from mechanistic insights that drive plant invasions.

There is mounting evidence showing that the ability to form successful mutualisms can be critical for successful plant invasions (Richardson *et al*. 2000; Traveset and Richardson 2011, 2014; Rodríguez-Echeverria *et al*. 2012; Le Roux *et al*. 2018) and range shifts (McGinn *et al*. 2018; Ramirez *et al*. 2019). We argue that insights on how lost mutualisms impact invasion success can inform us on how to circumvent problems involving beneficial microbial functions during crop domestication and breeding. To do so, we adapt here the conceptual framework of eco-evolutionary experience (EEE) often applied in invasion biology (Saul *et al*. 2013; Saul and Jeschke 2015) to crop plant domestication, breeding and selection. This framework provides a holistic view on the multiple ecological and evolutionary processes underlying biological invasions, which, we argue, should be considered to promote beneficial plant–microbial interactions in agriculture more successfully. Importantly, the EEE framework emphasizes the crucial importance of time and opportunities for adaptation that can only happen when symbiosis partners frequently interact (Saul *et al*. 2013). Prolonged contact between plants and microbial symbionts is important for reciprocal feedbacks to occur, whether ecological (i.e. increases and decreases in population size of the plant and/or microbial symbionts), or evolutionary (i.e. heritable genetic changes enabling reciprocal adaptation of the symbiosis partners, or adaptation by all the symbionts to new conditions) (McGinn *et al*. 2018; Dinnage *et al*. 2019).

**Figure 1.**
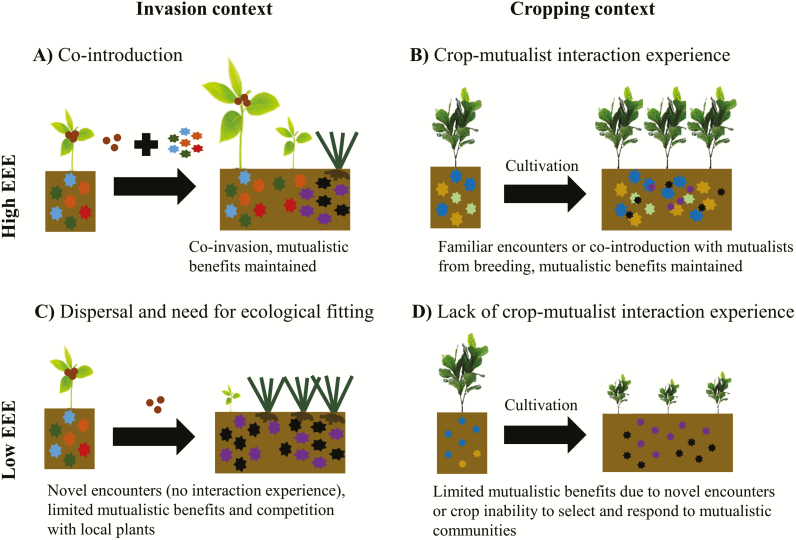
Analogy between the role of host plant–mutualist EEE during biological invasions and crop domestication. High EEE in biological invasions is here represented as the co-introduction scenario (A), in which plant and microbial propagules co-invade new areas. This allows the rapid establishment of familiar interactions in the new range that benefits establishment and spread of the population. The parallel situation in cropping is where crop and mutualists have been selected or bred together and cultivated in soils containing crop-adapted microbial mutualist communities (B). Low EEE in biological invasions is here represented as plant introductions without familiar mutualists, thus encountering barriers to establishment due to a lack in experience with novel soil biota (C). This implies time is needed for ecological fitting or adaptation to occur to increase the competitiveness of non-native plants against resident native plants. The translation to cropping is that genetic, phenotypic and ecological changes to crops during domestication and breeding have lowered their dependency and selectivity for microbial mutualists (D). When cropped in new areas, the inability to use mutualistic benefits from microbes limits their fitness. Different colours indicate different genotypes or taxa, while different symbol sizes refer to different sizes of the pools of infective propagules, i.e. microbial population sizes.

The potential loss of mutualistic functions following crop domestication discussed above could be looked at through the looking glass of EEE. Due to a narrowed gene pool and possible accumulation of deleterious mutations in symbiosis-related plant genes, modern crop cultivars are likely to have lost experience with their historically beneficial mutualistic microbial partners. This lack of interaction experience between crops and their beneficial mutualists may compromise mutualist-derived benefits under plantation conditions (Fig. 1).

Given its evolutionary underpinnings, the EEE concept can be integrated into crop development programs by having a closer focus on symbioses between the wild ancestors of modern cultivars and their microbial mutualists (Pérez-Jaramillo *et al*. 2018). We propose this can be done empirically by obtaining mutualistic microbial inocula from the wild ancestors of crops and subsequently cross-inoculation of modern cultivars and measuring their responsiveness in terms of nutrition, stress resistance and growth performance. The EEE framework enables verification of whether crops maintained the ability to benefit from ancestral microbial symbionts under a diverse range of abiotic conditions. Based on the performance of diverse crop genotypes grown under a range of soil and microbial inoculum conditions, the proposed framework can address whether symbionts will be beneficial under the variety of different environments in which a single crop is grown. Our experimental approach can also reveal whether symbiosis-related traits have been conservatively inherited from the crop’s wild ancestor(s), lost during domestication and through breeding, or newly acquired since domestication. An extensive application of this experimental approach across different crops would allow identification of crops that are most likely to benefit from root mutualisms (e.g. based on the domestication history, environmental preferences, extent of breeding measures). With the help of such approaches, the opportunities for EEE-conscious crop breeding and cultivation seem considerable, given available evidence from plant invasions that are facilitated by beneficial microbes.

In this viewpoint article, we emphasize and compare invasive legumes and legume crops, since (i) legumes rely heavily on eco-physiological flexibility conferred by symbioses with microbes (particularly mycorrhizal and rhizobial symbioses), (ii) legume productivity is highly dependent on cultivation practices, weather events, and edaphic and climatic conditions and (iii) compared to other crops, legumes have received less attention by breeders and therefore may have significant potential for improving yield and resilience to environmental unpredictability.

## Eco-Evolutionary Experience Is Important for Mycorrhizal and Rhizobial Root Nodule Symbiotic Functioning and Thus Should Be Fostered During Crop Breeding and Cultivation

Eco-evolutionary experience describes the historical exposure of an organism to biotic interactions over evolutionary timescales (Saul *et al*. 2013; Saul and Jeschke 2015). In the context of mutualistic plant–microbe associations, EEE stabilizes symbiotic associations by providing conditions that allow for symbiont filtering and adaptation, ultimately matching compatible symbiotic partners with the prevailing environmental conditions (Lau and Lennon 2012; Delaux *et al*. 2014). From a crop development perspective, EEE emphasizes the role of ancestral traits selected for in natural environments in driving the establishment success of crops under new plantation conditions (Gaut *et al*. 2018). Traits involving the interaction of crops with root microbial mutualists are those that should be viewed from an EEE perspective, but this has never been translated into cropping systems or the maintenance of supportive functions derived from microbial mutualists.

Cropping under edaphic and climatic conditions different from those during domestication and breeding is analogous to plant–mutualist co-invasions in novel environments, where symbiotic benefits mediate the success of plant establishment and spread (Fig. 1; Richardson *et al*. 2000; Le Roux *et al*. 2016). For instance, broadening of the realized ecological niche by mycorrhizae and rhizobia has not only facilitated plant invasion but also speciation under natural conditions (Rodríguez-Echeverria *et al*. 2012; Osborne *et al*. 2018). These mutualisms have also allowed the cultivation of grain and forage legumes (Weir *et al*. 2004) and pine trees in new geographic areas (Dickie *et al*. 2010, 2017). Although there is little information on EEE in crops, work on wild populations in natural habitats with rhizobia and mycorrhizal fungi offers analogues that can be translated to cropping systems.

### Eco-evolutionary experience between legumes and rhizobia

The idea that the EEE of plant–mutualist interactions can influence plant success in novel environments has been used to explain the invasiveness of Australian acacias (genus *Acacia*; Klock *et al*. 2016; Le Roux *et al*. 2017). In these systems, rhizobium genotype and acacia species identity have been shown to predict mutualistic benefits (Barrett *et al*. 2015), indicating co-evolution and specificity between interacting partners. Acacias have the highest establishment success when they co-invade new regions with their native rhizobia (i.e. co-introduction, highest EEE; Le Roux *et al*. 2017, 2018, Warrington *et al*. 2019). On the other hand, establishment success of acacias may also be facilitated by interactions with resident rhizobia found in the new range (i.e. ecological fitting, low EEE, Fig. 1C; Le Roux *et al*. 2018). Still, co-introduction is the most effective way for acacias to establish in new habitats over the short term (Warrington *et al*. 2019). Direct competition with resident plants for available mutualists seems to be driving the decreased success of invasions through ecological fitting. A major conclusion is that interaction experience between hosts and symbionts (i.e. co-introduction) results in higher fitness, which potentially involves strong positive feedbacks between co-invading partners that allow invading populations (or domesticated crops) to thrive (Fig. 1A and B; Dickie *et al*. 2017).

Unlike the acacias discussed above, many plants do not establish novel interactions with symbionts in new environments (Rodríguez-Echeverría *et al.* 2012; Traveset and Richardson 2014). This case of low EEE is analogous to crops being cultivated in new geographical areas in which host plants have no interaction experience with local soil biota (Fig. 1D). All symbiotic interactions fall along a continuum of specialization, and therefore successful establishment by specialized crop plants would be more reliant on co-introduction with their mutualists than by crops with generalist requirements (Fig. 1B; Toju *et al*. 2018). Plant–symbiont interactions are further compromised by the potential loss/alteration of symbiosis gene functionality during domestication and breeding, which reduces the capacity to establish partner-specific interactions (Martín-Robles *et al*. 2018; Sawers *et al*. 2018). Thus, symbiotic functions might be impaired under these circumstances due to either low partner specificity or the absence of historically beneficial microbial mutualists in new environments. These missing mutualists, however, may still be found in association with the crop’s wild ancestors (Pérez-Jaramillo *et al*. 2018).

### Eco-evolutionary experience in the plant–mycorrhizal symbiosis

Biological invasions also provide evidence for why plant–fungal symbioses need to be considered within an EEE framework and how this can inform crop plant success under novel ecological contexts (Dickie *et al*. 2017; Osborne *et al*. 2018; Urcelay *et al*. 2019). In montane forests of Central Argentina, native trees show elevational structure in plant–AMF associations, indicating some degree of partner specificity due to EEE (Urcelay *et al*. 2019). Urcelay *et al*. (2019) found invasive species along the same elevational gradient were equally responsive to AMF, suggesting successful ecological fitting between invaders and local AMF, i.e. low interaction specificity. In contrast, such ecological fitting has been unsuccessful for ectomycorrhizal (EM) tree introductions worldwide, which was attributed to the absence of effective resident mutualists in the new ranges. For example, the successful introduction of various pine and eucalypt species required co-introduction of their native EM fungi (Schwartz *et al*. 2006; Dickie *et al*. 2017).

Co-introduction is not a common pathway for plants and AMF. Production of fewer and bigger spores than EM fungi, and the comparatively limited extent of AMF mycelia are likely to limit the dispersal of these fungi across large geographic distances (Denison and Kiers 2011; Bueno and Moora 2019). For EM fungi, long-distance dispersal is known to occur via spores (Peay *et al*. 2012), and through soil mycelial networks at local scales (Thiet and Boerner 2007). However, a common feature across mycorrhizal groups is the sensitive asymbiotic life stage in soil after spore germination and before root colonization. This trait is likely to intensify the selection pressure of soil conditions on them. Relative to rhizobia, mycorrhizal symbioses are also much older and characterized by multispecies symbiotic microbiomes, which may explain why plant–mycorrhizal associations are generally less host-specific than legume–rhizobial associations. In sum, EEE seems more likely to influence the benefits and stability of the mycorrhizal symbiosis because of their seemingly stronger adaptation to the cropping soil conditions rather than specificity to host plants. Crop breeding will thus have to account for the cultivation conditions under which newly selected crop varieties are to be grown when breeding with mycorrhizas (Rillig *et al*. 2016).

### Mediating effects of soil nutrient availability on crop–mycorrhizal/rhizobial symbioses

It is becoming increasingly clear that soil biotic interactions causing plant–soil feedbacks cannot be isolated from the abiotic conditions within which they occur (Bennett and Klironomos 2019). The influence of abiotic soil conditions may be larger in mycorrhizal than rhizobial symbioses, as the former grows both in the soil and the root cortex, while the latter grows in specialized root/stem organs and is thus solely under the host’s control (Denison and Kiers 2011). Nevertheless, the mineral nutrient availability in soil indirectly affects the carbohydrate allocation to both groups via the host plant’s nutritional status. Hence, while exposure to different soils and host plants are expected to be important components of EEE in mycorrhizal symbioses (Bever *et al*. 2009; Rodriguez and Sanders 2015), for rhizobia, the host plant may act as the primary ecological and evolutionary filter.

Cropping practices (e.g. artificial selection) and high soil nutrient levels under cultivation and breeding are known to have compromised the nutritional and growth-promoting benefits of mycorrhizal and rhizobial symbioses (e.g. Hetrick *et al*. 1992; Kiers *et al*. 2007; Verbruggen and Kiers 2010; Martín-Robles *et al*. 2018; Sawers *et al*. 2018; but see Lehmann *et al*. 2012). For example, Martín-Robles *et al*. (2018) recently showed that various crops and their wild relatives responded differently to AMF inoculation. All of the 14 tested crop plants only responded positively to inoculation under low P availability, whereas the wild relatives responded positively independent of the soil P status. This suggests that breeding has altered the metabolic regulation of the mycorrhizal symbiosis in these crop plants (Martín-Robles *et al*. 2018). In addition, Sawers *et al*. (2018) pointed at the modification of root traits through breeding for pathogen resistance as an impediment for AMF root colonization and extraradical P acquisition regardless of nutrient availability.

For soybean–rhizobium symbiosis, Kiers *et al*. (2007) found modern soybean cultivars to have inferior sanctioning ability against ineffective rhizobia compared to old cultivars, suggesting that crop selection under high N availability has led to loss of host control and sanctioning. A 22-year N-fertilization experiment supported this notion, which found evolution of less effective rhizobia under high nutrient conditions in three *Trifolium* species, leading to reduced biomass production (Weese *et al*. 2015). Together these studies indicate that in legume crops, the consequences of EEE are subject to the cost-benefit balance of the mutualism from the crop’s side, which seem to more strongly affect rhizobial than mycorrhizal symbioses.

## From Theory to Practice: Testing the Functional Implications of Eco-Evolutionary Experience in Crop–Microbial Mutualisms Using Crop Wild Ancestors

Knowledge of EEE can inform us about the actual ability of modern legume cultivars to benefit from symbioses with mutualists from wild ancestors from a microbial community perspective. This can be achieved using cross-inoculation approaches similar to those used in plant–soil feedback experiments (Le Roux *et al*. 2018; McGinn *et al*. 2018; Gundale *et al*. 2019). With the increasing interest in the use of wild crop relatives to reintroduce traits that have been lost over generations of crop improvement (Schmidt *et al*. 2016; Dempewolf *et al*. 2017; Zsögön *et al*. 2018) and the availability of seed banks from ancestral genotypes, such experiments should become topical.

### How much EEE in crop-root mutualist associations has been retained during domestication, breeding and cropping?

To understand the contribution of plant-root mutualist EEE to improve crop–mutualist interactions, the experimental approach proposed here is based on microbial mutualist inocula of both wild relatives and modern cultivars and the inoculation of different modern crop genotypes (Box 1). The use of a variety of wild and cultivated soils and inocula parallels the invasion context in which non-native plants establish on soils with or without familiar symbionts, respectively. Like in the acacia invasions mentioned before, encountering inocula from a wild relative in the new range could facilitate spread due to partner matching through shared EEE. This hypothetical situation is a particular case of the ecological fitting scenario (Fig. 1C), one in which past plant–microbe EEE can facilitate rapid establishment.

Box 1. **An approach to experimentally determine the existence and importance of eco-evolutionary experience of modern crop cultivars to microbial symbionts.**

(A) Inocula of microbial mutualists are prepared from modern cultivars and crop wild ancestor(s) in an initial trapping or soil conditioning phase as in plant–soil feedback experiments by growing the respective plants in their soil of origin (arable or natural from the range of occurrence). Enriched and specific inocula can be obtained in the form of surface-sterilized roots and root nodules (in case of rhizobia).

(B) These inocula are then applied to modern crop cultivars grown in common garden-type experiments under sterilized and non-sterilized soil conditions. This design allows for inferences to be made on plant-symbiont compatibility and the effectiveness of ancestral symbionts in absence and presence of other soil biota. In *Treatments under common garden conditions*, we specify which ecological and evolutionary aspects of plant–microbe functioning are being tested under each treatment. In *Measurements*, we outline which features of plant growth responses, symbiotic functioning and evolutionary processes could be addressed with this experimental approach.

(A) Generation of mutualist inoculum by soil conditioning/symbiont trapping.

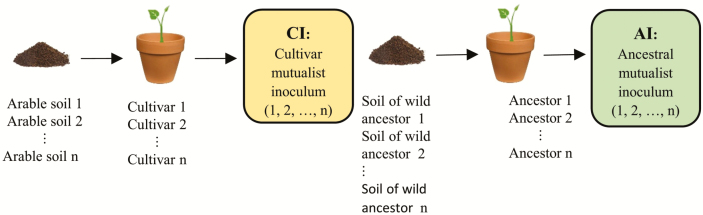

(B) Inoculation with microbial symbionts from the conditions under cultivation and the distribution range of the wild ancestor(s).

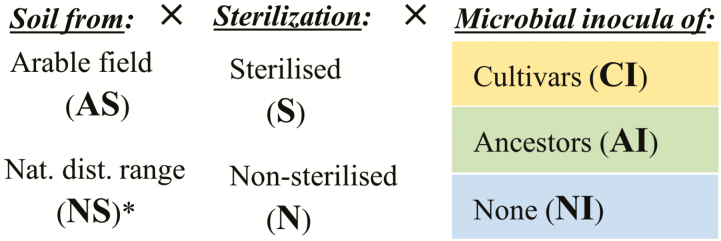

Treatments under common garden conditions (i.e. under identical edaphic and climatic conditions):

**Table:** 

Treatment	Testing for	Measurements
1) AS-S-CI	Newly acquired symbiosis traits in response to EEE with those symbionts becoming dominant after domestication.	*Yield components*: Total shoot and root dry matter; seed/grain number and weight; concentration of macro- and micronutrients.
2) AS-S-AI	Evolutionary conserved symbiosis traits inherited from the wild ancestor(s).	*Parameters of symbiotic functioning*: Number and weight of root nodules (for legumes); root colonization by AMF (particularly arbuscules and vesicles); stable isotopic signatures (^13^C, ^15^N); expression of symbiosis genes.
3) AS-S-NI	Adaptations to edaphic conditions. This is the control treatment for testing for adaptive traits to only microbial symbionts.	*Parameters of the individual symbiosis partners*: Genotypic and phenotypic differentiation (FST/QST); genetic diversity of the cultivars relative to the wild ancestor(s); presence/absence of symbiosis genes of the cultivars; genetic, phylogenetic and function-related diversity of the microbial symbionts based on phylotaxonomic (e.g. *nrDNA*, *recA*, *gyrB*, *glnII*) and functional DNA markers (e.g. *nif*, *nod*, *acdS*).
4) AS-N-CI	Positive plant–microbe–soil feedback upon repeated or continuous cultivation.	
5) AS-N-AI	The existence of symbiont selectivity or possible microbial symbiont recruitment limitation when in cultivation.	
6) AS-N-NI	The performance under current conditions. This is the reference of the *status quo*, the field-relevant control to test varieties on benefits from microbial symbioses.	

The six possible treatments with soil from the natural distribution range would indicate whether the abiotic and biotic soil conditions under arable land use are detrimental to the functioning of microbial symbioses. Therefore, these would indicate whether new land should be cultivated and/or measures taken to bring the abiotic and biotic conditions of arable soils back to those of the natural distribution range of the wild crop ancestor(s). However, because this would be a major undertaking and not in line with high output agriculture, this possibility is not further exemplified here.

**Figure 2.**
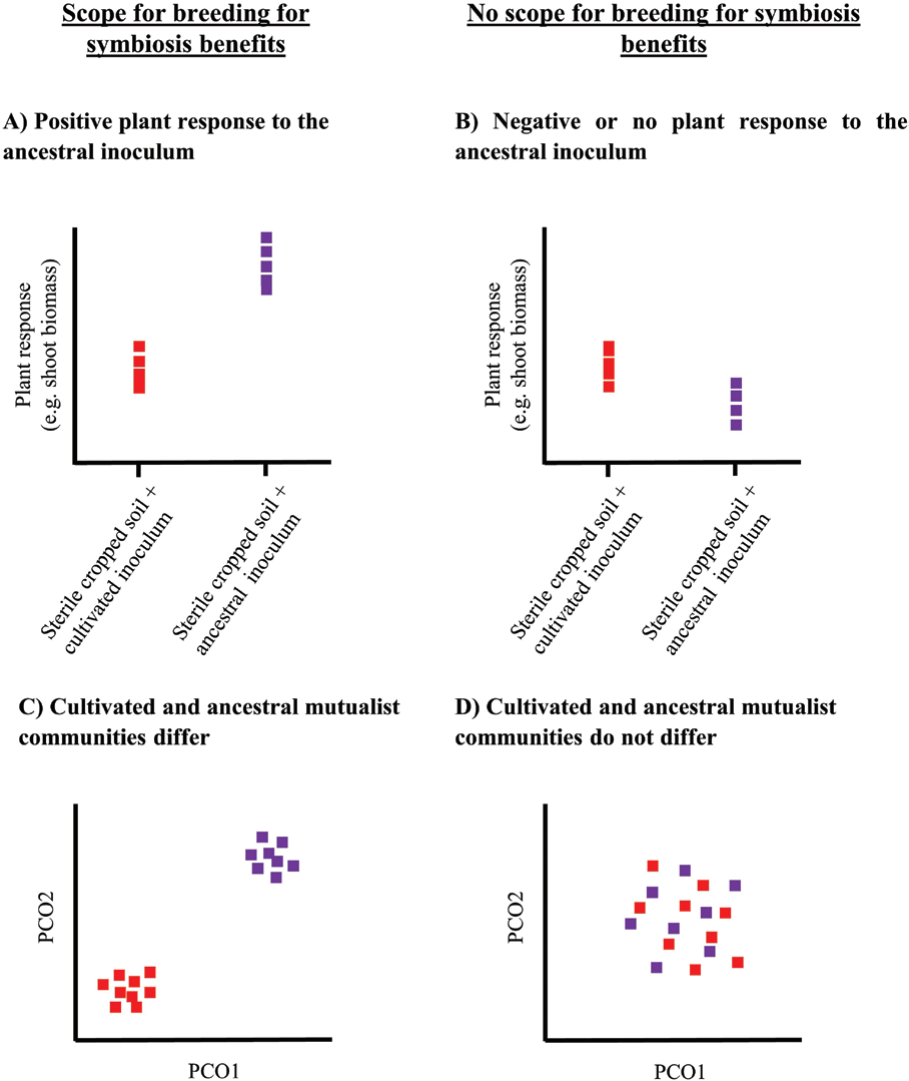
Visual representation of the relationships between plant functional responses and mutualist community similarities used to inform whether there is scope for breeding for symbiotic benefits. The approach is based on the inoculation of modern cultivars with microbial mutualists originating from wild ancestors and arable land. This reflects the degree of EEE between modern crops and their ancestral symbionts. Crop plant responsiveness to inoculation with microbial mutualist inocula from wild ancestors (purple squares) in comparison to inoculum from cultivated soil (red squares) can be used to predict whether breeding for symbiosis benefits can be considered. There is scope for breeding when plants inoculated with ancestral mutualists perform better than those inoculated with mutualists from arable land (A). This can be linked to differences in the composition and abundance of the mutualistic communities assembling in the roots, projected on a multivariate space (C). There is no scope for breeding for symbiotic benefits when there is a negative or no response to inoculation with ancestral mutualists (B), or when the cultivated and ancestral microbial mutualist communities do not differ (D).

As in trap culturing and the conditioning phase of plant–soil feedback experiments, inocula can be obtained by growing wild crop ancestors in their native soils and by isolating their root-associated microbial mutualists. These can be used to inoculate modern cultivars growing in sterile cultivated soils, as a way to test their ability to recruit and benefit from these putative ancestral symbionts (Box 1). Based on the experimental setup outlined in Box 1, the degree and strength of the effects of EEE can be estimated based on (i) the ability of modern cultivars to establish symbioses with ancestral mutualists and (ii) the ability of modern cultivars to benefit more from these associations than from those present in arable soils. This allows determining whether crop domestication and breeding have (i) compromised the genetic basis for beneficial plant–microbe interactions (i.e. symbiotic capacity), or whether (ii) the practices and conditions of cultivation impaired microbial symbiont survival and propagation (i.e. symbiont availability). Knowing whether a crop still relies on symbiotic services at all and whether these are derived from ancestral or novel associations can inform the development of inocula and of agronomic measures to promote crop-beneficial symbionts (i.e. symbiont management) (Fig. 2).

Both reciprocal symbiosis partner and symbiont-environment adaptations promoted by EEE are expected to increase the benefits plants reap from microbial symbionts. This is possible when genes modulating symbiosis specificity and effectivity have been preserved during crop domestication and breeding, manifesting when interactions with ancestral mutualists lead to better crop performance (i.e. selective microbial partner choice, optimized control over symbiosis functioning). With symbiont partner specialization comes predictable symbiotic associations (Toju *et al*. 2018), and this gives scope for the coordinated use of breeding and inoculation to optimize crop plant benefit from symbioses (Fig. 2A and C). Conversely, an inability to establish symbioses with ancestral mutualists may indicate that crucial symbiosis-related genes have been lost, or that deleterious mutations have accumulated, which impair symbiotic functioning. In this case, crop production could neither rely on nor take measures to make use of services provided by microbial symbionts (Fig. 2B and D), except if *de novo* domestication or genetic engineering are viable options (Quiza *et al*. 2015).

The most likely encountered scenarios are those in which symbiont selectivity and responsiveness to symbiosis have been decoupled. Crops may have switched to new mutualistic partners, being unable to take advantage of the benefits offered by their ancestral mutualists. Such changes in crop EEE can thus render active microbiome management unnecessary (Fig. 2B). Bread wheat represents an example of such changes in EEE under cultivation: positive responses to AMF returned after 30 years since its disappearance as a result of increased soil fertility by mineral fertilizers during the Green Revolution in the 1950s (Hetrick *et al*. 1992). At the same time, crops with geographically and ecologically restricted cultivation areas may persist because they have retained traits for highly effective symbiotic associations. The trade-off, however, is that only a small subset of microbial partners is available, and whose occurrence and prevalence could limit crop survival and yield at the long term. Managing the mutualistic microbial symbionts of such crop plants is very promising, since they are a clear functional target group to be co-distributed to new cultivation areas (Lemanceau *et al*. 2017). Examples suggesting the utility of such interventions are the joint introduction of European forage legumes and their root nodule symbiosis partners in Australia and New Zealand (Weir *et al*. 2004), or cultivation of pine trees in the southern hemisphere owing to co-introduction of compatible EM fungal symbiosis partners (Vellinga *et al*. 2009; Dickie *et al*. 2010).

## Conclusions

Despite a growing body of research on eco-evolutionary dynamics and their implications for plant–mutualist interactions in novel environments, relatively little attention has been paid to their role in crop development (Lemanceau *et al*. 2017). A consideration of crop-symbiont EEE is still insufficient during crop plant breeding and cultivation measures making use of available bioresources (Mariotte *et al*. 2018). Here, we translated the EEE framework of invasion ecology to crop–microbial root mutualisms. We emphasized the importance of considering EEE of crop wild ancestors and their beneficial microbes in plant breeding and cultivation to take better advantage of symbiotic services in the face of perturbation and change.

Current efforts to manipulate entire microbiomes (Busby *et al*. 2017; Toju *et al*. 2018) can also benefit from the EEE framework by assessing whether crop cultivars can associate with, and benefit from, whole microbial communities from their wild ancestors. Evidence from biological invasions indicates that plant–microbe co-invasion enables immediate fitness advantages, while invasion success can be constrained when introduced plants have to form, or adapt to, novel mutualists. This is a limitation that modern crops face in cultivation and that can impair their resilience to global change. To maintain high levels of crop–mutualist EEE, we need to promote conditions conducive for the stability of mutualisms (i.e. breeding under low-nutrient conditions, selecting cultivars with particularly beneficial mutualistic associations with AMF or rhizobia, managing the soil for high microbial diversity, involving the mutualists of crop wild ancestors in the selection process). While measures to account for EEE in cultivation may initially compromise crop yields, lasting and effective mutualisms in the face of environmental change seem indispensable for yield stability.
